# Common concepts in separate domains? Family physicians’ ways of understanding teaching patients and trainees, a qualitative study

**DOI:** 10.1186/s12909-015-0397-z

**Published:** 2015-06-27

**Authors:** Terese Stenfors-Hayes, Mattias Berg, Ian Scott, Joanna Bates

**Affiliations:** 1 Centre for Health Education Scholarship, University of British Columbia, Vancouver, BC Canada; 2 Department of Learning, Informatics, Management and Ethics, Karolinska Institutet, Stockholm, 171 77 Sweden; 3 Department of Emergency medicine, University of British Columbia, Vancouver, BC Canada

**Keywords:** Family medicine, Patient, Physician, Qualitative research, Trainee, Faculty development

## Abstract

**Background:**

Medical education is increasingly expanding into new community teaching settings and the need for clinical teachers is rising. Many physicians taking on this new role are already skilled patient educators. The purpose of this research was to explore how family physicians conceptualize teaching patients compared to the teaching of trainees. Our aim was to understand if there is any common ground between these two roles in order to support faculty development based on already existing skills.

**Methods:**

Semi-structured interviews with twenty-five family physician preceptors were conducted in Vancouver, Canada and thematically analyzed.

**Results:**

We identified four key areas of overlap between the two fields (being learner-centered; supporting the acquisition, application and integration of knowledge; role modeling and self-disclosure; and facilitating autonomy) and three areas of divergence (aim of teaching and setting the learning objectives; establishing rapport; and providing feedback).

**Conclusions:**

Finding common ground between these two teaching roles would support knowledge translation and inquiry between the domains of teaching patients and trainees. It would furthermore open up new avenues for improving training and practice for clinical teachers by better linking faculty development and continuing medical education (CME).

## Background

Around the world, medical education is expanding and moving into new and more distributed and dispersed communities. In Canada, where this study was set, the number of students entering MD programs increased by 73 % between 1995 and 2009 [1] and regional campus enrollment increased almost five-fold between 2005 and 2009 [1]. This expansion means that more regional community hospitals and community practice sites are now being used in undergraduate programs [2–5]. These new contexts of medical education, together with new models of education, such as longitudinal integrated clerkships, are increasingly relying on family physicians as preceptors. Between 2003 and 2007 the number of part-time family medicine faculty members in Canada increased by 64 % from 3605 to 5901. In the UK, approximately one third of general practitioners are now involved in teaching undergraduate medical students [6], and Australia is also projecting significant increases in community-based general practice preceptors [7].

The large recruitment of primary care preceptors [8–10] means that many physicians now find themselves cast into teaching roles because their practices are located at newly assigned community education sites. The need for qualified preceptors is often characterized as a major problem for expansion [11] and training to improve teaching skills has been recommended [12–17], especially for community-based preceptors [18]. This training is traditionally offered by units of faculty development linked to the universities. Ongoing primary care redesign is furthermore demanding new clinical skills of preceptors teaching in complex clinical settings [19]. In Canada and elsewhere, regular attendance at Continuous Medical Education (CME) activities is mandatory for all physicians. These activities address disease management, procedural instruction and practice management and may focus on various themes such as a certain diagnostics, optimized chronic disease management, and motivational interviewing. Physicians that need to improve their skills in patient education may choose appropriate CME activities to meet these needs. However, the number and distribution of teachers has increased the difficulty of providing and encouraging the attendance of family physicians at both faculty development and (CME) events.

Most newly recruited family practice preceptors are already experienced in teaching patients in the context of clinical care: They explain different treatment options to enable the patient to make treatment decisions; help patients understand possible diagnoses and warning signs of evolving symptoms (e.g., fever of unknown origin or abdominal pain); and provide support for self-management of chronic conditions (e.g., asthma or diabetes). As such, teaching and learning are central parts of the counseling interactions between physician and patient. Patient education and communication is also part of the undergraduate training to become a physician [20, 21]. It has been suggested that educating patients and trainees requires similar skills, and by learning to teach trainees, physicians may become better patient educators, and vice versa [22–24]. To our knowledge, no empirical data has yet been gathered to further explore or support this claim.

In this paper we explore how family physicians conceptualize key aspects of teaching patients and teaching trainees and how they identify the similarities and differences between them. The findings of our exploration may be used to explore the possibility of integrated faculty development and CME for physicians for improving their teaching of both trainees and patients simultaneously, and thereby helping to balance these competing learning needs [25]. We suggest that drawing upon identified similarities, as well as differences between the two fields will improve the effectiveness of training in both, as learning theory has shown that simultaneous exposure to a wider array of teaching contexts and situations improves learning [26]. Existing examples of integrated CME and faculty development do not integrate the learning in CME and the faculty development, but rather insert freestanding faculty development about separate issues into the CME event [27]. Clinical examples have successfully been used in faculty development for preceptors [28], but we are not aware of situations where the two types of teaching have been compared and contrasted. Comparing and contrasting is a central component of an effective learning strategy, but in order to draw interchangeably from experiences in patient counseling and clinical teaching, physicians must clarify their understanding of the similarities and also the differences between the two situations [26]. It requires an identification of existing knowledge and skills as well as the application of knowledge on a new topic to allow physician learners to discern the differences and similarities between the old and new [29, 30]: in this case teaching patients and teaching trainees. This study is a first step towards exploring this possibility.

## Method

This was an exploratory study, and as we wanted to focus on physicians’ conceptions of the phenomena rather than how their teaching is conducted, semi-structured interviews were deemed the most appropriate method. The research team consisted of two family physicians (IS and JB), one family medicine resident (MB), and one medical education researcher (TSH). The data collection was conducted in two phases. First, all the family physicians (*n* = 14) at a family practice teaching unit in British Columbia, Canada, were invited to participate in the study. The clinic provides teaching for undergraduate medical students and residents in family medicine. Information about the project was emailed to all physicians inviting them to participate. The study proposal was approved by the Research Ethics Board at the University of British Columbia and all respondents gave their written consent to participating. An initial interview framework was developed and subsequently modified based on two pilot interviews. Since the purpose of the interviews was to explore as many aspects of teaching patients and trainees as possible, most questions were followed by probing and follow up questions [31]. All interviews were conducted by TSH. For the second part of the data collection an email was sent out to all family physicians in the Greater Vancouver region of British Columbia who were engaged in the teaching of both patients and medical learners (*n* = 34). Twelve physicians were recruited and interviewed by MB using the same interview guide as in phase one. All interviews were audio recorded and transcribed. Saturation was determined through a preliminary analysis alongside data collection with discussion across the research team.

To ensure that we were not influenced by the description of teaching in one domain for the other, two researchers (IS and TSH) analyzed all the data from phase one independently from the perspective of teaching patients, and two researchers (JB and TSH) analyzed it from the perspective of teaching trainees. Each researcher read all transcripts, but after the first reading the transcripts were filtered to only include aspects of teaching patients or trainees respectively. The coding process was open and iterative. Classification of categories was independent of the frequency with which they were identified in the transcripts. As well as looking for convergence, we also sought negative instances to ensure that the analysis was not too aligned with our emerging notions of the findings. Each sub team met repeatedly and refined the coding for the two perspectives using constant comparison, where categories were further defined, merged, or deleted. We continued this process until we reached a negotiated consensus [32]. Once we had established one set of themes for teaching patients and another for teaching trainees, TSH synthesized these and extracted the similarities and differences. TSH did so through an iterative process during which JB and IS regularly provided feedback. The data from phase two was initially analyzed by MB using the same open and interpretive coding procedure as described above. Without having seen the findings of phase one, MB summarized the findings of phase two in a synthesized set of themes for similarities and differences for teaching patients and trainees. TSH reviewed the findings and based on the striking similarities between the two sets of findings the data was merged. As part of that process TSH reviewed and reanalyzed all the transcripts from phase two to strengthen the analysis. JB read all transcripts from phase two to verify the analysis.

## Results

Twenty-five physicians agreed to be interviewed: ten men and fifteen women, between the ages of 30 and 66 years. The respondents had been preceptors for 1 to 40 years. All respondents were family physicians, and some also had administrative roles in the medical education program. All respondents had regular opportunities to attend faculty development workshops at their campus aimed at improving their teaching of trainees and CME events focusing on patient communication and counselling. The extent to which the respondents had participated in these varied, but all were aware of the opportunities available to them. Preceptors spoke clearly and thoughtfully about their teaching of patients and trainees. We found areas of similarities as well as some differences in their conceptualization of the two teaching roles. To illustrate the findings, quotes have been selected from participants and include examples for both trainees and patients.

### Similarities

#### Being learner centered

Independent of whether the learner is a trainee or a patient, physicians try to adapt their teaching to the learner’s level of knowledge, what they believe they need to know, and a way of communicating that suits each learner.

I ask them what they know already, what they’ve read, what they’re comfortable with, what they’re not. I think it’s really important to ask what patients are thinking, what their concerns are [about patients].

To support the process of learning, physicians also emphasize the importance of patient/trainee motivation, and how they engage the patient/trainee if that motivation is missing. Some suggested that motivation among trainees to learn was often higher.

I think trainees tend to be engaged or keen. Whereas the switch is with patients, you need to engage them, you need to find out how motivated they are to learn about what’s going on with them.

Physicians also take the learner’s feelings into consideration and support their emotional health through reassuring patients. They do so by providing information regarding their disease that is appropriate to their needs and providing suggestions for further learning for the trainee to support their development as a physician.

It just feels like you just have to know where the person is, and if you don’t know that, then there’s no point [about trainees].

Physicians described how they incorporated cultural and social background and beliefs into their learner-centeredness in the case of patients, but less so with trainees. One respondent also differentiated between trainees and patients, in that tailoring of education to patients’ needs was more specific to their situation or context, whereas medical learner education was tailored more to their competency level.

With learners -- my teaching techniques vary very much depending on their level in their educational journey. Whereas with patients it very much depends on their situation at the time, their need to know, their desire to know and the emotional situation. It’s more of a situational as opposed to a level of competence that makes those decisions as to how you teach or approach.

### Supporting the acquisition, application and integration of knowledge

Physicians helped patients and trainees to gain new knowledge, contextualize information, and apply it to a specific situation. When teaching patients, physicians described how they translate the information the patient has retrieved from different sources (i.e. the Internet), help break down the information into ‘digestible chunks,’ provide the right information at the right time, and help the patient to contextualize and apply the information to their own situation. For trainees, physicians help them to contextualize their knowledge and apply it to a specific patient, and to integrate knowledge from textbooks and preclinical training with clinical experiences.

A translator of the information (that the patient has brought) and what that means to the patient. Working through what that is, or an interpreter or navigator [about patients].

For the students it’s teaching them about integrating their medical knowledge and using it to explore community resources and a realistic plan, not just a theoretical plan for investigation and management [about trainees].

Physicians accomplish this in the context of the patient or trainee (learner centeredness) as our first theme described, by building on the patient/trainees’ previous knowledge.

So we just have to recognize the level that they’re at and recognize where to challenge them [about trainees].

The tools of good communication are the same for both trainees and patients:

I think the principles of how you communicate with someone are the same: Thoughtfulness, respect, clarity, pitching things at the right level and inviting questions.

However, physicians expressed that the process of checking for understanding with the learner may be a fine balance to not intimidate them or to know whether they are answering honestly.

But I don’t know that the patients would honestly say, “No, I didn’t understand a word of what you said. It was way too complicated.” They’ll just say, “Oh, yeah, no, that make sense.” ‘Cause again, there’s a power dynamic and they don’t want to offend me. And there’s a social desirability bias and all that stuff at play [about patients].

I do try and get trainees to think about it (the answer to a question) first. And try and, in a way, you know, not to humiliate them or anything [about trainees].

Many use the same pedagogical approaches and teaching techniques (illustrations, case descriptions, Socratic questioning etc.) for both patients and trainees. One physician however suggests that with trainees one may be using more specific teaching techniques (such as quizzing) than with patients.

### Role modeling and self-disclosure

Physicians draw on their own actions and experiences to provide examples for both patients and trainees to learn from and to normalize patients’ and trainees’ experiences. With patients this role modeling and self-disclosure appears to also be about gaining legitimacy in the patient’s eyes and building rapport.

Sometimes I’ll say, “When my kids were sick, this happened.” Or, “I found with my kids with eating, this happened” and I think it helps the patient see that you understand the challenges that they’re having because you had similar challenges. So it’s normalizing their challenges. And by me saying, “I’m a parent and this is what I think is going on.” Or, “This is what I’m recommending,” it may help them feel more comfortable in the recommendation I’m making [about patients].

With trainees, the physicians also role model both as a person and as a professional with the role modeling being both explicit and implicit with a significant focus on work life balance, patient care, and lifelong learning techniques.

Sometimes I know the answer, but I say, “Let’s look this up,” because I want to model kind of-- let’s see if we can find this [about trainees].

I think the job description for physicians are that they need to be self-motivated. Continually assessing their own knowledge base and learning for the rest of their lives. And so I want to model that and sort of inspire them, you know [about trainees].

### Facilitating autonomy

Physicians showed support for the autonomy of both trainees and patients and strove to teach them how to manage their problem (whether their own illness or a patient problem) independently through techniques such as how to find relevant information and how to recognize when further help is needed. Physicians helped both patients and trainees to function “in the real world” (either by enabling patients to navigate successfully through the health system, or enabling trainees to practice medicine effectively in the community). Physicians described that the patients need to be able to manage their illness effectively on their own, while trainees need to experience autonomy in order to develop as independent physicians.

It’s the patient who needs to really identify what he or she needs. And it is really important to, again, that’s why it takes a bit of time, it’s to really try and understand what the needs are, help the patient articulate that and then help the patient sort of figure out what’s the best course of action [about patients].

You have to be comfortable letting go of the reins and allowing the residents to work through things [about trainees].

Let them figure things out, guide them through that sort of reasoning process and be there as a backup or a reassurance that nothing’s going to fall through the cracks. But allow them to experiment and figure out as best they can and learn from that process [about trainees].

### Divergence

#### Aim of teaching and setting the learning objectives

Physicians reported that in teaching both patients and trainees, knowing their objectives was core to good teaching. Physicians described the aims of teaching patients in terms of helping them to improve their health and quality of life and to slow the progression of disease. The patient thereby brings crucial expertise to the consultation as they are the experts in themselves and the way they experience their health. Physicians explained that with patients the focus of teaching is on the patient’s questions, interest and own perceived need to understand their disease or to change their behaviors and what they want to achieve. Physicians described the aims of teaching trainees quite differently: the trainees are there to learn to be physicians, which means that the physician is supporting their professional development and training them to be able to join a professional workforce. Thus, trainee learning outcomes need to align with formal and informal curricula, and licensing requirements.

I have expectations on trainees. I cannot demand patients to come up to certain level.

If the patient doesn’t want to know, they don’t have to know. They just have to know maybe to take their medication.

Many physicians seem to have a ‘gold standard’ for what they want the trainee to achieve and the physician more clearly sets the agenda and the learning outcomes for the trainees.

Although I know we’re supposed to teach clinical skills, I still think that it’s just so important for them to learn communication skills [about trainees].

### Establishing rapport

The importance of ‘connecting’ with patients was a central concept in teaching, as was establishing rapport and trust. Less importance was ascribed to connecting with trainees, while some physicians felt that in their work with trainees ‘a connection’ was established naturally through regular interaction. The importance that physicians attached to trainees connecting with patients also highlights the importance of the issue of physicians establishing rapport with patients.

So if you want your patients to become healthier and if you want them to live healthier lives, then you have to communicate with them and connect with them around where we’re going with this. And I think it has to be done together [about patients].

[On teaching the importance of establishing rapport]: I always look at it from the point of view of what did you learn from the patient. And if you just learn some facts you’re probably not going to get very far. Because you take, you know, a 60 year-old woman talking to a 25 year-old resident, this woman’s lived in the world for a long time; she’s not going to take the advice of a 25 year-old person unless that person has created an alliance.

Physicians often described trust as the core of a good relationship with a patient. One reason for the emphasis on trust was the patients’ freedom to choose another physician:

It’s the patient who has to approve you for them to stay with you. So I think probably the most important thing from their point of view is trust [about patients].

### Providing feedback

Physicians did not report giving overt feedback to patients, even if the physician had established clear objectives with a patient. However, giving constructive feedback was described as a central part of being a preceptor. Physician feedback to trainees was given in a variety of ways: upon request, during debrief after a patient encounter, or spontaneously, for example during a case presentation.

Recognizing their strength and weaknesses and being able to reflect back on what they’ve done and say, this is what’s changed, this is what hasn’t changed, this is what you’ve learned [about trainees].

Some physicians did report feeling hesitant to provide negative feedback to trainees.

We’re pleasers and we don’t always want to criticize trainees when they’re not doing a particularly good job [about trainees].

### Reciprocal learning

Physicians described their relationships with both patients and trainees as rewarding and satisfying. With patients the longitudinal aspects were especially cherished as many followed their patients and their families for 30 years or more. With trainees the ability to follow their professional growth was also valued. Additionally, physicians expressed that there was reciprocal educational value in what they could learn from their medical trainees.

It’s always surprising what you learn from residents. I always manage to learn something from them [about trainees].

The other big value is trainees keep you up to date. They ask you questions and they give you some confidence that you’re maintaining your abilities [about trainees].

## Discussion and conclusions

Our analysis shows examples of both similarities and differences in the way physicians conceptualized their teaching of patients and trainees. We found four key areas of overlap: first, we identified learner-centeredness as a key concept. Physicians described this orientation with both trainees and patients in similar ways, such as focusing on understanding the trainee or patient as a whole person with individual needs and expectations to be integrated in the learning situation as described. This perspective has been described in both medical education and patient education literature [33, 34]. Our second similarity is that with both trainees and patients, physicians tried to facilitate the acquisition, integration, and application of knowledge as well as emphasize the importance of providing information at the right time. Both medical education and patient counselling have developed theoretical frameworks that outline the process of learning in their domain. In the higher education literature, the concept of teaching in the zone of proximal development describes the importance of understanding what the trainee knows and does not yet know, and focusing the teaching in the zone just beyond their current knowledge [35]. In the patient education literature, behavioral change models emphasize the process that patients undergo when moving through changing their health habits [36]. These models include the patient’s readiness for change, and the importance of presenting information when the patient is ready to use it. Theoretical frameworks in use in both domains use the key concepts of assessing the learner’s readiness to learn, and ensuring that the right level of information is presented and support provided to the learner at the right time. Although the language is different, there are clearly similarities between theoretical frameworks in use in the two domains.

Physicians were aware of their implicit role modeling with trainees and patients. With trainees, the physicians also used explicit role modeling. Such role modeling has been shown to support trainee learning and development [37, 38]. However, role modelling is not only about clinical competence as one respondent suggests but also about good teaching ability and personal attributes [39]. Physicians also used self-disclosure to provide insights for their learners drawn from the physician’s experience and to help build rapport with the patient and to normalize the patient’s challenges. However, the value of self-disclosure as a clinical tool is not clear [40, 41]. Finally, we also found that physicians aimed to support the autonomy of both trainees and of patients. Autonomy is also a way of enhancing self-efficacy [42], which is the belief an individual holds regarding her ability to succeed in a specific situation [43]. In clinical education, new models focus on trainee initiative and emphasize self-directed and self-regulated learning to help develop independency [44–46]. In patient education, self-management and self-care are key concepts [47]. Self-management can be supported by patient empowerment which focuses on their right and ability to choose by and for themselves [48]. Although under different names and techniques, the aim in both clinical education and patient education is to increase the learners’ ability to ‘manage on their own’. We believe that the areas identified by the respondents are well in line with what is valued in medical trainee education today.

One area of difference we identified was that when teaching trainees, physicians take a much more active role in setting the objectives for learning. This may be related to the perceived difference in the goals of each group. With patients, the goal of teaching was to improve patient health and well-being, and patient health behaviors, with patients naturally bringing greater knowledge of their illness experience to the patient-physician consultation. With trainees, the goal of teaching was to facilitate trainee development into competent physicians (i.e. ‘the gold standard’). While physicians have more expertise in what it means to be a competent physician than the trainees they are teaching, the emergence of competency based curricula may shift this divergence, as trainees may take on a larger role in determining their own needs to achieve a set of competencies.

Our second area of difference was related to building rapport and trust and this differential was linked to the importance of establishing a ‘connection’ with patients. The physician’s ability to establish rapport also affects the level of trust established [49, 50] with both trainees and patients. With patients, continuity of care facilitates the development of a trusting relationship [51]. Effective teaching of trainees also requires such a relationship [52–54]. The difference between these two relationships is that the physician has the power of expertise over both trainee and patient but the physician also holds legitimate power over trainees in the formal assessment of them [55]. Our interview findings reflect the emphasis on the ‘connection’ between patient and physician, whilst the importance of a similar connection with trainees was not described by the physicians. The importance of establishing such relationships is increasingly emphasized in medical education [56] and supported through the development of longitudinal clerkship programs [57] and mentor programs [58]. These new programs demonstrate the importance of continuity of relationship between preceptors and learners [59]. However, none of the respondents in the current study were participating in longitudinal medical education innovations which may be a reason for the lack of emphasis among them in this field.

The third area of divergence focuses on the role of feedback in learning. Physicians we interviewed spoke at length about feedback to trainees but never mentioned feedback to patients. Clinical teaching models emphasize the need for constructive feedback as a learning tool, and many efforts in both assessment and faculty development are aimed at promoting effective feedback to trainees [60–63]. If feedback is such a learning tool for trainees, conceptualization of physician feedback on patients’ learning and behavioral change might improve the development of their self-management skills. However, it may be that physicians use other techniques such as tracking of health indicators such as blood pressure, weight, or blood sugar to provide feedback and to achieve the suggested changes. Our findings also showed how educating patients and trainees is considered rewarding, but in somewhat different ways. For both types of teaching the physicians enjoy the longitudinal aspects of following growth and development, whether personal or professional. With trainees the physicians also felt they learned a lot from the trainees regarding current medical practice.

### Limitations of study

This study is based on interviews with family practice preceptors from one university, with various clinical practices. We did not explore differences among physicians with respect to the level of the trainees they usually supervised, i.e. if they were undergraduates or residents. This may be an interesting question to pursue in further research. All respondents had extensive experience in teaching both trainees and patients and most of them had also practiced and taught in more than one setting. The response rate for the study was high in phase one and data saturation suggests that the number of interviewees was acceptable. Because phase one of our research took place in a family medicine academic teaching unit, we might expect these physicians to be thoughtful about teaching in both domains. Our second dataset, however, indicates that these findings hold true in other contexts. Trustworthiness of the findings has been strengthened by local and international presentations and discussions of the results [64, 65].

### Implications for practice

The need for improved teaching skills among physicians has been advocated by many, yet the difficulty of providing family physicians both faculty development and CME events has increased. We therefore propose a merging of these two domains: teaching patients and trainees. We suggest that some aspects of faculty development be reframed to make better use of physicians’ existing knowledge and experience drawn from patient care and to facilitate transfer of knowledge and skills from one domain to the other. Thus we can imagine supporting physician CME at faculty development events and supporting faculty development at CME events. However, in order to draw interchangeably from experiences in patient counseling and clinical teaching, physicians must understand the similarities and also the differences between the two situations [26].

Faculty development activities are highly valued by the participants [66] and may lead to personal growth and for participants to become more critically reflective teachers [39]. Perhaps this increased level of reflective teaching can be transferred to patient education as well? We can anticipate that increased knowledge about the similarities and differences of the two domains may lead to new understanding of each of these domains. An increased awareness on the importance of building rapport with trainees can be created from our experiences in patient education, and we can bring what we know about feedback into our patient encounters. Finally, our study also showed that being a preceptor was considered an important way to maintain one’s medical knowledge.

With our work we propose a way of approaching faculty development to meet the needs of increasing numbers of preceptors in community-based settings. We would like to invite discussion regarding how a more synergistic understanding of the teacher role (of both trainees and patients) may lead to an increased capacity for teaching the next generation of physicians as well as benefit patient care. In this paper we have explored how family physicians conceptualize their teaching of trainees and patients respectively. While the language and context in the two domains (teaching patients and teaching trainees) may differ, some of the underlying concepts and meanings are similar. We believe that an increased integration of the overlapping aspects of the two domains of teaching patients and trainees in the training of physicians and others may gradually lead to the emergence of a shared language, and may help advance the education, research, and practice of physicians independently of whether the learner they are seeing is a trainee or a patient.
